# The Role of Tumor-Associated Macrophages in Hematologic Malignancies

**DOI:** 10.3390/cancers13143597

**Published:** 2021-07-18

**Authors:** Emanuele Cencini, Alberto Fabbri, Anna Sicuranza, Alessandro Gozzetti, Monica Bocchia

**Affiliations:** Unit of Hematology, Azienda Ospedaliera Universitaria Senese, University of Siena, 53100 Siena, Italy; fabbri7@unisi.it (A.F.); sicuranza4@unisi.it (A.S.); gozzetti@unisi.it (A.G.); bocchia@unisi.it (M.B.)

**Keywords:** TAM, leukemia, lymphoma, myeloma, prognosis

## Abstract

**Simple Summary:**

Tumor-associated macrophages (TAM) represent a leading component of the tumor microenvironment in hematologic malignancies. TAM could display antitumor activity or, conversely, could contribute to tumor growth and survival, depending on their polarization. TAM are polarized towards form M1, with a pro-inflammatory phenotype and an antineoplastic activity, or M2, with an alternately activated phenotype, associated with a poor outcome in patients presenting with leukemia, lymphoma or multiple myeloma. The molecular mechanisms of TAM in different types of hematologic malignancies are different due to the peculiar microenvironment of each disease. TAM could contribute to tumor progression, reduced apoptosis and angiogenesis; a different TAM polarization could explain a reduced treatment response in patients with a similar disease subtype. The aim of our review is to better define the role of TAM in patients with leukemia, lymphoma or multiple myeloma. Finally, we would like to focus on TAM as a possible target for antineoplastic therapy.

**Abstract:**

The tumor microenvironment includes dendritic cells, T-cytotoxic, T-helper, reactive B-lymphoid cells and macrophages; these reactive cells could interplay with malignant cells and promote tumor growth and survival. Among its cellular components, tumor-associated macrophages (TAM) represent a component of the innate immune system and play an important role, especially in hematologic malignancies. Depending on the stimuli that trigger their activation, TAM are polarized towards form M1, contributing to antitumor responses, or M2, associated with tumor progression. Many studies demonstrated a correlation between TAM, disease progression and the patient’s outcome in lymphoproliferative neoplasms, such as Hodgkin lymphoma (HL) and non-Hodgkin lymphoma (NHL), even if with conflicting results. A critical hurdle to overcome is surely represented by the heterogeneity in the choice of the optimal markers and methods used for TAM analysis (gene-expression profile vs. immunohistochemistry, CD163vs. CD68vs. CD163/CD68 double-positive cells). TAM have been recently linked to the development and progression of multiple myeloma and leukemia, with a critical role in the homing of malignant cells, drug resistance, immune suppression and angiogenesis. As such, this review will summarize the role of TAM in different hematologic malignancies, focusing on the complex interplay between TAM and tumor cells, the prognostic value of TAM and the possible TAM-targeted therapeutic strategies.

## 1. Introduction

The innate immune system defends the host from infections and non-self antigens in a non-specific manner; thus, it could be involved in the identification and destruction of neoplastic cells [1,2,3]. It includes natural physical barriers, the complement system, natural killer (NK) cells, mast cells, dendritic cells, neutrophils, eosinophils, basophils, monocytes and macrophages. Macrophages represent a leading component of the innate immune response, are involved in the inflammation, tissue repair and phagocytosis of pathogens and can release pro-inflammatory cytokines and chemokines [4,5,6]. Phagocytosis is a mechanism leading to the destruction of foreign antigens; macrophages can secrete cytokines that promote the recruitment of leukocytes from the circulating blood to the sites of inflammation. The antitumor effect of macrophages includes the killing of cancer cells through both their own Fc receptors that bind antibodies on the surface of cancer cells and the production of nitric oxide (NO) and tumor necrosis factor (TNF). After phagocytosis, macrophages can expose tumor antigens on their surface, thus permitting the antigen recognition by T-lymphoid cells [4,5,6].

Since the 1990s, immunohistochemical (IHC) analyses have shown many macrophages in the tumor microenvironment (TME) [7,8]. This infiltration could be induced by tumor cells by secreting molecules such as chemokine ligands with a C-C motif (CCL) or C-X3-C motif (CXCL) and granulocyte–macrophage colony-stimulating factor (GM-CSF) [9,10,11,12,13]. A high number of macrophages has been frequently observed in both solid and hematological malignancies, and it was first considered as a mechanism involved in anticancer surveillance [14,15]. However, several reports demonstrated that the so-called tumor-associated macrophages (TAM) could conversely contribute to oncogenesis and neoplastic progression as “bad guys”, by promoting genetic instability and angiogenesis, while reducing the immune response and apoptosis [4,16,17,18].

**Figure 1:**
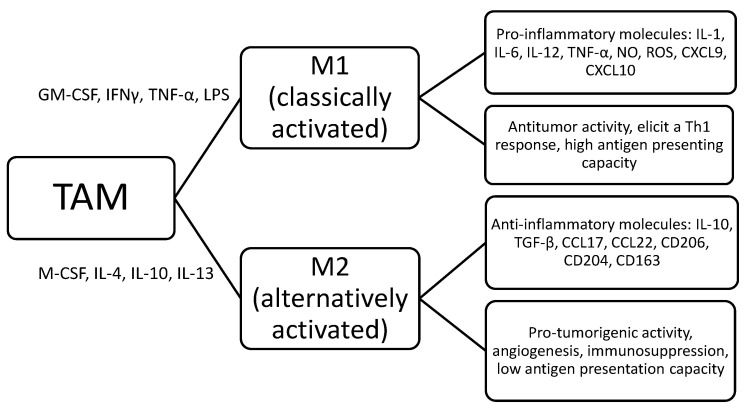
Tumor-associated macrophages polarization model in hematologic malignancies. TAM, tumor-associated macrophages; GM-CSF, granulocyte and monocyte colony stimulating factor; IFN, interferon; TNF, tumor necrosis factor; LPS, lipopolysaccharide; M-CSF, monocyte colony stimulating factor; IL, interleukin; TNF, tumor necrosis factor; NO, nitric oxide; ROS, reactive oxygen species; CXCL, chemokine ligands with C-X3-C motif; Th, T helper; TGF, tumor growth factor.

**Figure 2:**
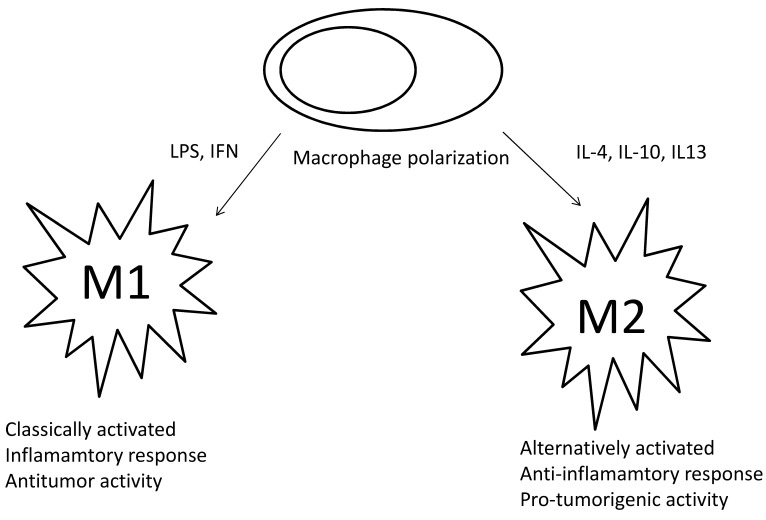
Visual mean of tumor-associated macrophages polarization.

TAM are characterized by a wide morphological, phenotypical and functional heterogeneity [4]. Complex interactions have been reported between reactive cells, including TAM and malignant cells in patients with leukemia, Hodgkin lymphoma (HL), non-Hodgkin lymphoma (NHL) or multiple myeloma (MM); as a final result, TAM could acquire an immunosuppressive and oncogenic phenotype [19]. Macrophages are characterized by remarkable plasticity and were initially divided into six subtypes (immunosuppressive, angiogenic, metastasis-associated, invasive, activated and perivascular), depending on the stimuli that trigger their activation [20]. However, in the currently accepted classification, even if oversimplified, TAM have been divided into M1 (classically activated) and M2 (alternatively activated), probably representing the 2 extremities of a continuous spectrum [21,22,23]. Due to this different polarization, which occurs in the tissue, M1 TAM could provoke a Th-1 response and play an antineoplastic effect leading to tumor suppression, while M2 TAM, with a low antigen-presenting capacity, could promote tumor growth and survival by inducing angiogenesis and immunosuppression [24]. In particular, as shown in Figure 1 and Figure 2, the M1 subtype, triggered by GM-CSF, bacterial products and interferon-γ, could secrete pro-inflammatory molecules, such as interleukin (IL)-1, IL-6, IL-12, IL-23, TNF-α, NO, CXCL9, CXCL10, CXCL11 and reactive oxygen species; conversely, the M2 subtype is activated by IL-4, IL-10, IL-13 and express anti-inflammatory molecules, such as IL-10, tumor growth factor (TGF-β), CCL17, CCL18, CCL22, mannose receptor C type 1 (CD206), class A scavenger receptor (CD204) and hemoglobin scavenger receptor (CD163), which are currently used as M2-associated markers [21,22,23,24].

In order to exert their action within the tumor microenvironment, TAM have to be recruited and then polarized into a tumor-promoting phenotype. TAM recruitment is mediated by chemokines, such as CCL2, CCL5, CCL7 and CXCL1, macrophages(M) CSF and vascular endothelial growth factor (VEGF) [25,26,27,28,29,30,31]. At the tumor site, a complex interplay occurs between TAM, malignant cells and stromal cells, in which stroma-derived and tumor-derived factors, such as IL4, IL-10, IL-13 and TGF-β, cause a shift towards M2 polarization [32,33,34,35]. Once recruited and polarized, TAM could contribute to tumor progression, metastasis and chemo resistance by producing matrix remodeling molecules and impairing innate or adaptive immune cells functionality [36,37,38].

This review summarizes the publications associated to the role of TAM in hematological malignancies, including HL, NHL, MM and leukemia, with a particular focus on M2, which are mainly related to tumor progression and treatment response.

## 2. Materials and Methods

We performed a computerized search in MEDLINE in order to find full-text publications, written in English, focusing on relationship between TAM and hematologic malignancies. The key terms to search included “tumor-associated macrophages OR TAM OR M1 macrophages OR M2 macrophages OR CD68 OR CD163 OR CD204 OR CD206 AND hematologic malignancies OR Hodgkin lymphoma OR Hodgkin disease OR non-Hodgkin lymphoma OR multiple myeloma OR acute myeloid leukemia OR acute lymphoblastic leukemia OR acute T-cell lymphoma/leukemia OR chronic lymphocytic leukemia”. We have also searched in the reference list of selected articles in order to perform a more comprehensive research. We included retrospective studies but excluded conference abstracts and case reports. For each study, we have extrapolated: disease, methods of TAM determination, TAM markers, the antibodies and the choice of cut-off for high vs. low infiltration, patient number, treatment schedule and the relationship between TAM and disease outcome, especially progression-free survival (PFS) and overall survival (OS).

## 3. TAM Role in Leukemia

Due to the growing evidence that leukemic cells interact with bone marrow (BM) reactive cells, including macrophages, the concept of leukemia-associated macrophages has been formulated [39,40]. A possible correlation between an immunosuppressive microenvironment and leukemia progression has been hypothesized for acute lymphoblastic leukemia (ALL), adult T-cell leukemia/lymphoma (ATLL), acute myeloid leukemia (AML) and chronic lymphocytic leukemia (CLL) [41]. The pathophysiology could be linked at least in part to an immune escape, due to a reduced ability of macrophages to recognize antigens and to perform their phagocytosis [39,40,41,42,43].

### 3.1. Acute Lymphoblastic Leukemia

ALL is a rare and aggressive disease, often characterized by a poor outcome despite high-dose therapy and allogeneic stem cell transplantation (allo-SCT) [44]. Even if there has been an undeniable therapeutic advance in the last few years, OS remains significantly reduced compared to the age-matched general population. A higher white blood cells (WBC) count at diagnosis (reflecting an elevated tumor burden), an older age and comorbidities (related to a reduced treatment tolerability) are recognized as prognostic factors for a lower complete remission (CR) and shorter duration of response (DOR) [44]. However, many other immunophenotypic, cytogenetic and molecular disease-related factors have been studied and there is a growing interest about a possible prognostic relevance of an immunosuppressive microenvironment [45,46,47,48].

#### 3.1.1. Pre-Clinical Studies

In a mouse model of T-ALL with Notch1 overexpression, a different gene expression profile (GEP) between BM and splenic macrophages was observed [49]. After coculture, splenic macrophages caused greater leukemic cells stimulation compared to BM macrophages [49]. Moreover, ALL cells could release the bone morphogenetic protein 4 (BMP4), as a mechanism to induce the M2 polarization of TAM, which are in turn capable of producing CCL2, IL-6 and IL-10 [50]. As discovered recently, the deletion of the stromal interaction molecule (Stim) 1 and 2 in ALL cells could reduce TAM infiltration and favor a shift towards an M1 polarization through IFN-γ [51].

The concept of a BM niche as a sanctuary supporting ALL progression was recently shared, in which TAM acquire immunosuppressive properties from the leukemic cells, such as a reduction in their phagocytic activity by the interaction of signal regulatory protein (SIRP)α with CD47 molecule located on the leukemic cells surface [52].

#### 3.1.2. Clinical Studies

The results obtained from pre-clinical studies helped to develop clinical studies in ALL patients; however, the hypothesized prognostic role of TAM in ALL has not been demonstrated in clinical studies, even if macrophages count in patients’ samples was increased compared to healthy subjects. In a retrospective study, BM biopsies of 52 B-ALL patients and 14 healthy controls were analyzed [53]. In B-ALL BM, a decreased proportion of M1 TAM and CD27-positive T-cells was reported, while M2 TAM and myeloid-derived suppressor cells (MDSC) were increased. The author suggested the reactive BM microenvironment in ALL cases significantly differs from healthy controls [53]. In another study, 97 BM samples of acute leukemia patients (26/97 with ALL) were compared to 30 with iron-deficiency anemia (IDA), as healthy control group [47]. In leukemic BM, CD68-positive, CD163-positive and CD206-positive macrophages count was significantly increased compared to IDA and significantly decreased after therapy in patients achieving a CR, even if it remained higher than in the control group. If we consider CD68 as a pan-macrophage marker, CD163-positive or CD206-positive/CD68-positive ratio was increased in the BM leukemic samples compared to the control group, further supporting an M2 polarization [47].

### 3.2. Adult T-Cell Leukemia/Lymphoma

ATLL represents an uncommon neoplasm, linked to the human T-lymphotropic virus type 1 (HTLV-1) and characterized by dismal prognosis [54]. The incidence is higher in Japan, the Caribbean and sub-Saharan Africa, with older age at the onset in cases outside of Japan. Anthracyclines-containing regimens demonstrated limited efficacy, and a significant proportion of patients were refractory or relapsed after first-line therapy [54].

#### 3.2.1. Pre-Clinical Studies

In ATLL lines, a significant cell activation was noted by direct coculture with M2 TAM. The authors showed some soluble factors were implicated in this interaction between M2 TAM and tumor cells, such as TNF-α, C5a and IL-6 [55]. Moreover, a higher CD163 expression was induced by direct TAM–ATLL cells interaction and ATLL cells proliferation was reduced if cocultured with CD163-silenced macrophages [55].

The number of CD204-positive TAM was associated with malignant cells proliferation, measured according to the Ki-67 labeling index, giving a novel insight into ATLL pathophysiology [56].

#### 3.2.2. Clinical Studies

The role of TAM in ATLL showed in pre-clinical studies was confirmed and a significant correlation between CD163-positive TAM and poor prognosis was reported in 58 ATLL patients, in which double-immunostaining demonstrated CD163-positive TAM also expressed a CD68 marker [55]. In the univariate analysis, a higher number of both CD68-positive and CD163-positive cells was significantly associated with a dismal disease outcome; however, in the multivariate analysis, only an elevated CD163 expression confirmed a prognostic correlation [55]. Interestingly, CD206-positive macrophages showed an overlap in ATLL tissues with CD163-positive elements, but not with the CD204-positive population, further reaffirming a remarkable plasticity [56].

### 3.3. Acute Myeloid Leukemia

AML represents an aggressive malignancy due to the BM clonal expansion of myeloid precursors. Circulating tumor cells (blasts) in the peripheral blood occur together with anemia, neutropenia and thrombocytopenia [57]. Adverse prognostic factors include an advanced age, high WBC count at diagnosis, history of myelodysplastic syndrome (MDS) or chronic myeloproliferative neoplasm (MPN), cytogenetic alterations such as deletion of chromosome 5 and/or 7 and molecular aberrations such as Fms-related receptor tyrosine kinase 3 (FLT3) mutations [57].

#### 3.3.1. Pre-Clinical Studies

Several reports showed TAM could influence AML cells survival and drug resistance. The frequency of BM CD163/CD206 double-positive M2 TAM was analyzed and was increased in AML patients compared to healthy donors [58]. AML cells could polarize BM and splenic TAM towards a pro-leukemic phenotype in mouse models. The growth factor independence 1 (Gfi1), a transcriptional repressor involved in myeloid and lymphoid hematopoiesis, plays a fundamental role in TAM polarization. Gfi-deficient mice showed an increased production of pro-inflammatory molecules, thus Gfi1 could enhance in vitro M2 polarization induced by IL-4 and reduce M1 polarization induced by LPS [58].

Similarly, peritoneal macrophages in mixed lineage leukemia (MLL)-AF9-induced AML mouse models showed a prevalent M2 TAM phenotype. The results of this study suggested the AML microenvironment significantly influenced the morphology, killing and phagocytic function of peritoneal macrophages; peritoneal TAM could acquire an M2 phenotype with different GEP compared to normal macrophages [59]. Consequently, a reprogramming towards an M1 polarization could exert an immunotherapeutic effect against AML. Another study investigated the molecular basis for different TAM subtypes; in leukemia models, BM TAM showed M1 characteristics while splenic TAM had more M2 characteristics [60]. An interferon regulatory factor 7 (IRF7) gene could contribute to M1 TAM polarization through SAPK/JNK pathway activation and showed a higher expression in BM TAM compared to splenic TAM [60].

#### 3.3.2. Clinical Studies

Pre-clinical studies suggested the capability of M2 TAM to influence AML progression and drug resistance, and this was confirmed by clinical data. Due to the fact that AML cells could polarize TAM towards a pro-tumor subtype, it seems necessary to eliminate these TAM subsets by depletion and/or reprogramming towards an antitumor subtype. It could have a prognostic relevance, as shown in an interesting study, in which the clinical impact of TAM in AML patients was investigated using an open database called *BloodSpot* [60]. A higher expression of CD163 was associated with poor prognosis in human AML, while no correlation with survival was noted for CD68, further suggesting that M2 TAM could be related to a dismal disease outcome, rather than total macrophages count.

In the above-mentioned study including both ALL and AML, CD163/CD206 double-positive TAM had an increased expression in leukemic samples than in healthy donors [47]. The M2 marker CD206 was identified as a novel prognostic factor for AML patients using the algorithm CIBERSORT [43]. The authors confirmed an increased M2 TAM frequency compared to healthy donors;this was correlated with a poor outcome. Specifically, high CD206 expression was associated with reduced event free survival (EFS) and OS in AML cohorts. Interestingly, among 175 AML cases presenting with intermediate-risk cytogenetics, 3-y EFS and OS for patients with low and high CD206 expression were 47% vs. 25% and 56% vs. 32%, respectively (*p* < 0.001) [43].

Lastly, a low level of monocytic leukemia zincfinger protein (MOZ) was associated with reduced M1 polarization and poor prognosis in AML cases, and miR-223, capable of suppressing M1 polarization, was involved in MOZ regulation [61].

### 3.4. Chronic Lymphocytic Leukemia

CLL is the most common leukemia subtype in western countries and in elderly patients and is characterized by highly variable clinical course and prognosis. Approximately one-third of CLL patients do not require any therapy and have a normal lifespan. However, a not negligible proportion exhibit aggressive behavior that requires treatment at diagnosis, or develop a clinical progression after an initial indolent phase [62]. Biological variables, such as cytogenetic aberrations and immunoglobulin heavy chain variable region genes (IGHV) mutational status, represent the most important factors to consider before treatment choice [63]. The highest risk group is represented by CLL harboring TP53 mutations and/or del(17p), in which targeted therapy is strongly recommended as first-line regimen [64,65].

#### 3.4.1. Pre-Clinical Studies

The first identification of reactive cells in the CLL microenvironment that could promote CLL survival in vitro was performed more than 20 years ago, with the identification of the so-called nurse-like cells (NLC) [66]. Subsequent investigations showed NLC were CLL-associated macrophages and were therefore considered as TAM [67,68]. NLC present a prevalent M2 polarization, with high CD163 and CD206 expression [69,70,71]. NLC functions include recruiting cells, enhancing CLL proliferation and inhibiting the apoptotic pathways [66,67,68,69,70,71]. Specifically, NLC can support CLL cells growth and survival through CXCL12, CXCL13 and vascular cell adhesion molecule 1 [66,67]. In a mouse model, the migration inhibitory factor was identified as a cofactor for disease pathogenesis by favoring macrophages accumulation in the organs involved by CLL [69,70]. An in vivo deep crosstalk between NLC and CLL cells was discovered and a macrophage depletion could reduce CLL engraftment and prolong mouse survival [72]. Antineoplastic activity could be based on interfering between leukemic cells and NLC interactions by an IFN-mediated NLC reprogramming or by the inhibition of the CSF1 receptor [73,74].

#### 3.4.2. Clinical Studies

Despite the findings of pre-clinical data about the NLC support of CLL cells, a prognostic role for TAM/NLC has not been demonstrated in a clinical cohort to date, even if a TAM-based clinical approach is under investigation [75,76].

Ibrutinib, a Bruton tyrosine kinase (BTK) inhibitor, is widely used for CLL therapy, both as first-line regimen and for relapsed/refractory (R/R) cases [77,78]. Ibrutinib could inhibit the macrophage production of CXCL13 and directly modify NLC phenotype and functions by targeting BTK expressed on NLC [79]. Surprisingly, ibrutinib could favor M2 polarization and could represent a possible explanation for reduced drug efficacy [80,81].

Lenalidomide, an immunomodulatory and antiangiogenic agent clinically active in CLL patients, could improve NLC phagocytic activity and reduce NLC survival support to CLL cells through a shift towards a pro-inflammatory NLC phenotype [82,83].

## 4. TAM in Multiple Myeloma

MM is the second most common hematological plasma cells malignancy and accounts for 1–2% of all cancers, with an increasing annual incidence of approximately 4.5–6.0 new cases/100,000 inhabitants in Europe [84]. In the last few years, the use of novel agents has given an unprecedented increase of response and survival improvement; however, most patients relapse, need multiple lines of treatment and eventually die from the disease or its complications [85].

### 4.1. Pre-Clinical Studies

The interaction between MM plasma cells and TME is well established, including osteoblasts, osteoclasts, mesenchymal stem cells (MSC) and TAM [86]. TME influences the homing, survival, proliferation, drug resistance and immune escape of MM plasma cells, which in turn can recruit macrophages through chemotactic molecules and alter the BM TME by suppressing cytotoxic effects of reactive immune cells [86]. Specifically, TAM carry out a fundamental role in MM pathogenesis, including promoting BM plasma cells homing and proliferation, angiogenesis and the so-called vasculogenic mimicry, further supporting MM immune evasion and progression [87,88,89,90,91,92,93].

MM plasma cells in vitro could favor an M2 TAM polarization by upregulating CD206 expression of cocultured macrophages [94]. In MM cell lines, CD68-positive TAM represent an important component of TME and could inhibit the drug-induced apoptosis of neoplastic cells by the cleavage of caspase-3 and poly-ADP ribose polymerase (PARP) [92,95]. Moreover, P-selectin glycoprotein ligand-1 (PSGL-1) and intracellular adhesion molecule-1 (ICAM-1) on the MM cells surface could induce TAM activation and were involved in TAM-induced chemo resistance through the Src, Erk1/2 and c-myc pathway [96]. In addition, together with MSC, TAM could support MM cells survival and proliferation through IL-6 and IL-10 [96]. An early in vitro study showed a coculture with macrophages could increase the MM cells growth rate, and ex vivo matured human macrophages could increase human MM cells proliferation by producing IL-6, IL-10, IL-12 and VEGF [96,97,98].

Due to the TAM production of insulin growth factor (IGF)-1, clodronateliposome-mediated macrophage depletion was capable of inhibiting MM development in vitro and in vivo by influencing plasma cells migration and homing to the BM [99]. As demonstrated in several reports, during progression from monoclonal gammopathy of undetermined significance (MGUS) to MM, M2 TAM could also favor the angiogenic switch by secreting VEGF [87,88]. In a recent paper, in mouse models, BMI1, a polycombgroup protein, showed the capability to modulate the pro-myeloma functions of TAM, which showed higher BMI1 levels compared to normal macrophages. In a BMI1 knockout model, a reduced TAM proliferation was reported, together with a reduced expression of angiogenic factors [93].

De Beule and colleagues showed the pro-tumor effect of TAM was correlated with Stat3 pathway activation in 5T33MM cells. Interestingly, AZD1480, an ATP-competitive Janus kinase (JAK)2 inhibitor, could inhibit this effect and resensitize MM cells to bortezomib [100,101]. Lastly, micro (mi)RNA were investigated as potential factors contributing to MM molecular pathogenesis; in this field, exosome-derived miR-let-7c and miR-214 were recently involved in M2 TAM polarization and angiogenesis promotion in BM TME [102,103].

### 4.2. Clinical Studies

Pivotal clinical studies confirmed both the promising pre-clinical data and the prognostic relevance of both total and M2 TAM, in which CD68/CD163 double-positive M2 TAM were associated with an increased micro-vessel density and reduced survival, independently of the tumor stage [104,105,106,107,108].

In the BM of MM patients with an active disease, CD206-positive M2 TAM were increased compared to healthy subjects or patients presenting with MGUS [94,100].

CD163 expression detected by IHC was assessed in 198 MM patients treated with bortezomib-based regimens. A high CD163-positive M2 TAM expression at diagnosis, with the used cutoff of >55/high power field, was associated with a lower CR rate and worse PFS and OS, and its value was confirmed in a multivariate analysis [104]. Furthermore, an elevated level of soluble M2 TAM markers CD163 and CD206 was associated with a reduced OS, while a higher M1 density was correlated with an OS improvement [91,105].

In a retrospective study, 68 MM patients were enrolled, and TAM were assessed with anti-CD68 and anti CD163 antibodies [106]. An elevated CD68-positive and CD163-positive TAM expression had a detrimental effect on 6-y OS in a multivariate analysis. As a complementary finding, an elevated microvessel density was associated with an increased CD163-positive TAM number, further suggesting M2 TAM could have an adverse prognostic role [106].

In a relevant study, the polarized functional status of BM TAM by CD68, inducible NO synthase (iNOS) and CD163 IHC staining was investigated in 240 MM patients [107]. A reduced overall response rate (ORR) was observed in patients presenting with a high CD68-postive and CD163-positive TAM density; however, only high CD163 expression was associated with inferior PFS and OS. CD163 and iNOS were identified as independent prognostic factors and were combined with the international staging system (ISS) in order to generate a new prognostic score [107].

As angiogenesis induction is one of the mechanisms through which M2 TAM favor MM progression, M2 TAM infiltration and the correlation with the pro-angiogenic factor CD147 were evaluated in a spectrum ranging from MGUS to relapsed MM [108]. CD163 was used as M2 marker and the used cutoff for M2 infiltration was 100 per core. The authors showed a significant OS reduction for relapsed MM patients with high M2 expression (32 vs. 6 months, *p* = 0.02), further suggesting a prognostic role for CD163-positive TAM in MM [108].

Andersen and colleagues evaluated CD163 as a soluble protein in 104 blood samples and 17 BM samples in newly diagnosed MM cases [91]. CD163 expression was higher in BM compared to blood samples and was associated with a higher ISS stage and other adverse prognostic factors. An elevated CD163 expression, with the suggested cutoff of 1.8 mg/L, was correlated with a poor outcome, further indicating TAM could influence MM growth and progression [91].

## 5. TAM in Hodgkin lymphoma

Classic Hodgkin lymphoma (cHL) is a highly curable lymphoid malignancy that mostly affects young adults.

CHL is characterized by good prognosis in most cases, in which neoplastic cell, called Reed–Sternberg (R–S) cells, are B-cells with a defective phenotype [109]. In HL samples, R–S cells represent a minority of total cellularity and are surrounded by a reactive TME, including eosinophils, basophils, dendritic cells, plasma cells and TAM [109]. First-line therapy is represented by an ABVD regimen (doxorubicin, bleomycin, vinblastine and dacarbazine) for a total of 4–6 cycles, followed by radiation therapy as consolidation for early-stage disease. Despite encouraging results, approximately 20% of total cases experience R/R disease and none of the published prognostic factors at diagnosis can definitely identify patients at high risk of treatment failure [109].

### 5.1. Pre-Clinical Studies

Due to the peculiar HL biology, a possible prognostic role of TAM was first suggested in 1985 [110]. After a direct interaction with HL cells, TAM could induce in vitro HL cells proliferation through STAT3 pathway activation [111]. The signaling events and the mechanism of M2 TAM polarization in HL is not well understood and further research is needed in this field.

Programmed death protein 1 (PD-1) and its ligands (PD-L1 and PD-L2) represent fundamental molecules for the immunoevasion of R–S cells in HL. PD-L1 is expressed by R–S cells and TAM in the TME, while PD-1 is expressed by reactive T-cells. Carey and colleagues found PD-L1-positive TAMs could surround R–S cells and could especially interact with PD-1-positive/CD4-positive T-cells, further suggesting CD4-positive T-cells represent the target of the PD-1 blockade [112].

Interestingly, the CD163-positive monocytes population expressing PD-L1 was more elevated in HL patients compared to diffuse large B-cell lymphoma (DLBCL) [113]. Consistently, in HL, CD163/PD-L1/PD-L2 gene expression was more elevated compared to DLBCL. The phenotype could revert to normal after the ABVD regimen and the monocytes depletion from the baseline blood samples of HL patients could favor CD3-negative/CD56^hi^/CD16-negative NK-cells activation [113].

Recent reports demonstrated the role of PI3K inhibition on M2 TAM polarization, further suggesting the PI3K-Akt pathway is involved in HL pathogenesis, and its blockade could favor a switch towards an M1 polarization and lead to tumor regression [114].

### 5.2. Clinical Studies

Unlike in leukemia, for HL, pre-clinical studies are reduced compared to clinical data and there is not a strong correlation between pre-clinical and clinical findings. TAM in HL, determined both by GEP and by IHC using the markers CD68, emerged as a relevant prognostic factor of disease-specific survival and PFS in the pivotal paper by Steidl and colleagues, outperforming the international prognostic score (IPS) [115]. These macrophages could be considered as “bad guys”, with a pro-tumor activity and a possible inhibition of drug-mediated cell death, leading to a reduced treatment response, disease progression and reduced survival [115]. Since then, many subsequent reports were published, but showed conflicting results about a definitive association between TAM and survival, as illustrated in Table 1 [115,116,117,118,119,120,121,122,123,124,125,126,127,128,129,130].

Azambuja and colleagues showed a lack of IHC reproducibility compared to GEP, while other reports suggested CD163 could represent a more appropriate marker to detect TAM infiltration compared to CD68, that could stain myelomonocytic cells, fibroblasts and endothelial cells [115,116,117,118,119,120,121,122,123,124,125,126,127,128,129,130].

It was also speculated that TAM could represent an expression of reactive TME detected by 18F-FDG PET [117,118,119,120]. A correlation between high TAM infiltration at diagnosis (CD68 > 25%), PET positivity (Deauville score 4–5) after two ABVD cycles and reduced PFS and OS has been observed in a small study; however, these findings were not confirmed by three larger studies performed in recent years [117,118,119,120].

Recent reports demonstrated HL patients with the highest M2 TAM count, measured using CD163 or CD68/CD163 as M2 polarization markers, had a significantly reduced disease-free survival (DFS), PFS and OS, further confirming M2 TAM could support tumor progression and immune escape [122,123,128,129].

Even if a metanalysis confirmed the prognostic value of both CD68 and CD163 in HL cases, the main hurdles to overcome are represented by the choice of TAM markers (CD68 vs. CD163 vs. CD68/CD163 double-positive), the different cutoffs used to define a high TAM expression and the different monoclonal antibodies used for IHC (KP-1 or PGM-1 for CD68 evaluation) [101,131].

## 6. TAM in Non-Hodgkin Lymphoma

DLBCL and follicular lymphoma (FL) represent the most common aggressive and indolent NHL subtype, respectively. FL has an excellent prognosis and therapy should be reserved to patients with B symptoms, cytopenia, bulky disease or rapidly progressive disease. First-line treatments include obinutuzumab (O) or rituximab (R) in association with cyclophosphamide, doxorubicin, vincristine and prednisone (CHOP) or bendamustine (B) [132]. Conversely, DLBCL is an aggressive disease and a significant proportion of cases are refractory or relapse early after R-CHOP is used as first-line therapy [133].

### 6.1. Pre-Clinical Studies

TAM M2 polarization could be induced by apoptotic NHL cells, and M2 TAM have a reduced galectin-3 expression, a glycoprotein involved in the clearance of apoptotic cells progression, finally leading to NHL progression [134]. Even if the prognostic value of CD163 as an M2 marker is debated in FL, a high CD163-positive TAM density was associated with neovascularization located in the interfollicular area [135]. Early in vitro studies demonstrated macrophages could induce NHL cells proliferation after a direct contact. In this field, TAM could produce cytokines such as C5a, IL-6 and TNF-α, that in turn activate Stat3 and NF-kB pathways [136]. Lastly, in DLBCL, M2 TAM could contribute to extracellular matrix remodeling through legumain, that leads to fibronectin degradation and enhances local angiogenesis [137].

### 6.2. Clinical Studies

As for HL, there are limited pre-clinical studies for NHL cases and clinical data showed conflicting results compared to pre-clinical findings. Pivotal GEP-based studies demonstrated that TME has a prognostic relevance in DLBCL. A stromal-1 signature, characterized by genes expressed by components of an extracellular matrix, was associated with a good outcome, while a stromal-2 signature was characterized by a dismal prognosis, mainly due to increased angiogenesis [138]. Moreover, several studies showed TAM CD68-positive content was associated with an increased survival in DLBCL, while an elevation of CD163/CD68 ratio, suggestive of M2 polarization, was predictive of a dismal outcome, as illustrated in Table 2 [139,140,141,142,143,144,145,146,147,148]. Riihijarvi and colleagues described the outcome correlation of GEP-assessed CD68 mRNA levels and IHC CD68 protein expression. A cohort of 59 cases was investigated using the anti-CD68 KP1 antibody and an arbitrary cut-off choice; CD68 was associated with a good prognosis in patients receiving rituximab in combination with chemotherapy, while the disease outcome was poor in patients receiving only chemotherapy [139]. In this paper, CD163 expression in GEP and IHC did not demonstrate any prognostic value. Another study investigated 165 patients treated with R-CHOP, in which high CD68 expression was associated with an improved OS, while both PFS and OS were decreased in patients presenting with an elevated CD163/CD68 ratio [140]. Conversely, the other report did not observe any correlation between CD68 and survival.

As suggested for HL, a wide heterogeneity in the choice of IHC antibodies, TAM markers, scoring methods (manual or automated) and threshold could generate these conflicting findings. As suggested by a recent meta-analysis of 11 studies, the highest hazard ratio for survival is reached by CD163/CD68 TAM ratio, further confirming M2 TAM represent a robust outcome predictor for NHL patients, including DLBCL and FL [149].

In FL, high CD68-positive TAM expression was correlated with a poor clinical outcome in several reports, but other studies showed opposite findings [150,151,152,153,154]. Interestingly, a high CD163 TAM count was correlated with increased angiogenesis and microvascular density, which were in turn linked to a poor disease outcome.

## 7. Macrophages and Bioenergetic Modifications

One of the major aspects of the tumor–stromal interaction that was observed in solid and hematologic malignancies is represented by the bioenergetics dependence–independenceinteraction. In this field, a reverse Warburg effect, metabolic coupling and hypoxic induction, and even a mitochondrial transfer, have been suggested as possible mechanisms of interaction between tumor cells and stromal cells in leukemia, lymphoma and MM. Although the reactive cells involved in this crosstalk that finally supports tumor growth and survival have been generally identified as stromal elements, we have to mention a possible relationship between macrophages and bioenergetics modifications. In the Philadelphia (Ph) chromosome-positive leukemic cells, in which the BCR–ABL oncogene is constitutively expressed as a fusion protein with tyrosine kinase activity, the therapeutic concentration of the tyrosine kinase inhibitor imatinib reduced glucose uptake by suppressing glycolytic cell activity [155]. Furthermore, the mitochondrial Krebs cycle activity was improved, finally resulting in an increased energy state and apoptosis. The author suggested imatinib, without direct cytocidal activity, could reverse the Warburg effect in BCR–ABL-positive cells by switching from glycolysis to mitochondrial glucose metabolism [155]. The Warburg effect was demonstrated in leukemic–stromal cocultures and was mediated by mitochondrial uncoupling associated with the uncoupling protein activation 2 (UCP2). Under exposure to normal oxygen concentration, a significant interaction between leukemic cells and BM-derived MSC was reported, which promoted the accumulation of lactate in the culture medium without glucose consumption, with a subsequent reduction of mitochondrial membrane potential in both cell types. Interestingly, in leukemic cells, this reduction was mediated by mitochondrial uncoupling and was associated with increased UCP2 expression [156].

Overall, in hematologic malignancies, there is a strong association between cellular metabolism, mitochondrial bioenergetics and the interaction with supportive TME, finally contributing to drug resistance. Lymphoma and CLL cells could increase mitochondrial biogenesis with the aim of adapting to oxidative stress. Conversely, in MM, changes in bioenergetics represent an adaptive response to drug-induced stress [157]. In the BM niche, a reverse Warburg effect has been recently demonstrated, in which metabolic changes occurring in stromal cells could provide support to adjacent tumor cells. Interestingly, an increased mitochondrial biogenesis of tumor cells due to the acquisition of new mitochondria transferred by MSC could increase adenosine triphosphate production by oxidative phosphorylation and could mediate drug resistance [157]. This field could represent a possible rationale for a future target therapy for R/R patients with hematologic malignancies, who represent unmet medical need.

## 8. New Perspectives and Possible TAM-Related Treatment Approach

Since TAM are relevant for cancer progression, they could represent a target for immunotherapy. The aim to treat hematologic malignancies using M2 TAM as a target is under investigation with at least four research fields, including the blockade of monocyte recruitment, TAM depletion, TAM reprogramming into M1-phenotype and molecular signaling modification, as illustrated in Table 3.

Since the CCL2–CCR2 signaling axis is involved in monocytes trafficking, its inactivation in solid neoplasms significantly reduced tumor growth. Trabectedin, a DNA-binding sea squirt-derived compound, approved to treat solid tumors, could kill monocytes and macrophages and exert an antiangiogenic activity by inhibiting CCL2 and VEGF production [158,159]. In CLL mouse models, trabectedin showed an antileukemic role by reducing TAM recruitment, directly depleting TAM and increasing the memory T-cells count [158]. Due to a triggered apoptosis in MM cell lines, cell cycle arrest, VEGF depletion and NK cells upregulation, a possible role has been proposed for trabectedin in MM [159].

CSF-1R signaling is another important pathway for TAM recruitment and differentiation [74,160,161,162]. It was demonstrated that CSF-1R inhibition could block TAM polarization in T-ALL mouse models [160]. A treatment combination between vincristine and a CSF-1R inhibitor could improve survival in leukemic T-ALL mice compared to vincristine as a singleagent [160]. These findings were confirmed in CLL, in which pacritinib, a JAK2/FLT3 double inhibitor, blocked CSF-1R and was associated with TAM depletion and an improved survival [74]. The antineoplastic effect of CSF-1R inhibition was recently observed in ATLL, in which it could reduce tumor growth and sensitize malignant cells to chemotherapy [161]. Interestingly, idelalisib, a PI3K inhibitor approved for CLL and FL therapy, could block the CSF-1-mediated TAM spreading [162].

Since CXCR4/CXCL12 axis could promote M2 TAM polarization, it has been speculated that its inhibition could permit TAM reprogramming towards a M1-phenotype. Interestingly, in recent preclinical studies, the addition of the CXCR4 inhibitor plerixafor, currently used to mobilize stem cells, with ruxolitinib and venetoclax improved survival in T-ALL [163].

Ph chromosome-positive B-ALL cells could be reprogrammed in vitro into normal macrophage-like cells after exposure to myeloid differentiation-promoting cytokines. This lineage reprogramming could eliminate the leukemogenicity of Ph-positive B-ALL cells, thus representing a promising future treatment strategy [164].

Biphosphonates, currently approved for MM with bone lesions, could deplete TAM and abrogate MM establishment in vivo [99]. In hepatocellular carcinoma, the transformation into M1 pro-inflammatory TAM could be induced by sorafenib, an FLT-3 inhibitor, arguing it could represent a complementary mechanism of action for the drug when used to treat FLT3-positive AML [171]. The intermediate CD40 agonist ChiLob 7/4 and the weak agonist dacetuzumab, which were capable of inducing pro-inflammatory cytokines, gave encouraging results in pivotal trials, including hematologic malignancies [165]. M1 TAM polarization could represent a possible mechanism of action of IFN-α, giving an explanation of the reported efficacy as a preemptive therapy for ALL patients after allo-SCT [166]. Artesunate (ART) induced an increase in inflammatory monocytes in vitro, while it reduced macrophage expression of CD206 and CD163. After contact with monocytes reprogrammed by ART, the in vitro apoptosis of leukemic cells was increased, due to ART changing the monocyte phenotype by JAK2/STAT3 downregulation [167].

Lenalidomide, an antineoplastic agent with a pleiotropic effect, could influence TME through the improvement of T- and NK-cells function in combination with a significant reduction of angiogenesis [168]. Moreover, in mouse MM models, lenalidomide could permit a M2 TAM depletion and a reduction in IL-10 production [172].

Cladribine, a purine analog, currently approved to treat hairy cell leukemia with variable treatment modalities, inhibits in vitro the secretion of pro-inflammatory cytokines and phagocytosis in human M1 macrophages activated by LPS [173,174].

The most promising research field is probably represented by the usage of CD47, a glycoprotein commonly expressed in both myeloid and lymphoid malignancies, as a treatment target. The CD47 binding to its receptor SIRPα inhibits macrophage phagocytosis and favors the immune escape of tumor cells. Preclinical studies demonstrated CD47 inhibition reduced ALL and MM cells growth and improved T-cell-mediated cytotoxicity [169].

The pivotal phase I study by Advani and colleagues investigated the Hu5F9-G4 antibody and the possible synergism with rituximab in 22 R/R DLBCL or FL cases (95% were rituximab-refractory). Due to the rarity of dose-limiting effects, a phase II study was initiated; ORR was 50% (CR rate was 36%) [170]. After a median follow-up of 6.2 months and 8.1 months for DLBCL and FL, respectively, the treatment response was maintained in 91% of responsive patients. These findings demonstrated a promising antineoplastic activity by blocking the so-called CD47 “don’t eat me” signal [170].

## 9. Conclusions

Targeting the innate immunity to treating hematologic malignancies represents an attractive strategy, due to the peculiar TME of leukemia, myeloma and lymphoma and the well-established crosstalk between neoplastic and reactive cells. We have summarized the critical TAM role in tumor growth and progression, especially for M2 TAM, which is characterized by an immunosuppressive phenotype. We can suggest that high baseline M2 TAM content, especially in association with clinical prognostic factors, could contribute to the identification of patients characterized by high-risk disease at diagnosis. A TAM-target strategy could act in association with chemoimmunotherapy and reduce drug resistance; thus, we suggest investigating this promising approach in future studies focused on poor-risk patients.

## Figures and Tables

**Table 1:** Clinical studies about prognostic role of tumor-associated macrophages (TAM) in Hodgkin lymphoma.

Reference	Number of Patients	Marker	Antibody	Cut-Off	Survival Correlation
Steidl et al. [115]	166	CD68	KP1	Steidl score (<5%, 5–25%, >25%)	Inferior PFS, DSS
Azambuja et al. [116]	265	CD68, CD163	KP1 10D6	Steidl score	No
Cencini et al. [117]	200	CD68	KP1	Steidl score	No
Agur et al. [118]	98	CD68	PGM1	Steidl score	No
Cuccaro et al. [119]	102	CD68	PGM1	Steidl score	Inferior PFS
Touati et al. [120]	158	CD68	PGM1	<25%, ≥25%	Inferior PFS, OS
Kayal et al. [121]	100	CD68	(CD68/G2)	Steidl score <25%, >25%; <12.9%, >12.9% (the quartiles) <18.2%, >18.2% (median)	No No No
Klein et al. [122]	88	CD68, CD163	KP1 10D6	<25%, >25%; <25%, >25%	No Inferior OS
Yoon et al. [123]	144	CD68, CD163	KP1 10D6	<20%, >20% <20%, >20%	Inferior EFS, DSS, OS Inferior EFS, DSS, OS
Tan et al. [124]	287	CD68, CD163	KP1 10D6	<12.7%, >12.7% 16.8% (CD163)	Inferior FFS, OS Inferior FFS, OS
Greaves et al. [125]	90	CD68	KP1	<5%, 5–15%, >15%	Inferior FFTF, OS
Jakovic et al. [126]	52	CD68	PGM1	<25%, ≥25%	Inferior EFS, OS
Kamper et al. [127]	288	CD68, CD163	KP1 10D6	<7.8%, ≥7.8% <21.1%, ≥21.1%	Inferior EFS, OS Inferior EFS, OS
Werner et al. [128]	76	CD68, CD163	PGM1 10D6	<724, 725–937, ≥938 <769, 770–1325, ≥1326	Inferior DSS Inferior DSS, OS (NS)
Karihtala et al. [129]	130	CD68, CD163	Ab955 Ab188571	Continuous variable Continuous variable	Inferior OS No
Mohamed et al. [130]	81	CD68	Ab955	<20%, 20–50%, >50%	Inferior DFS, OS

Abbreviations: PFS, progression-free survival; DSS, disease-specific survival; OS, overall survival; EFS, event-free survival; FFS, failure-free survival; FFTF, freedom from treatment failure; NS, not significant.

**Table 2:** Clinical studies about prognostic role of tumor-associated macrophages (TAM) in diffuse large B-cell lymphoma.

Reference	Number of Patients	Marker	Antibody	Cut-Off	Survival Correlation
Riihijarvi et al. [139]	59	CD68, CD163	KP1 10D6	26/hpf Various	Better PFS, OS (R-chemo) No
Marchesi et al. [140]	61	CD68/CD163	Doublestain (NS)	double-marked cells/ total macrophages > 0.2	Inferior DFS, OS
Wada et al. [141]	101	CD68 CD68/CD163	PGM1 Doublestain (G2 EnVision)	<average, >average <average, >average	Inferior OS Inferior OS
Nam et al. [142]	109	CD68 CD163 CD68/CD163	PGM1 10D6 Doublestain (automated system Ventana)	Best cut-off Best cut-off Best cut-off	Better OS Inferior OS Inferior OS
Hasselblom et al. [143]	176	CD68	KP1	<median, >median	No
Coutinho et al. [144]	161	CD68	KP1	<median, >median	No
Gomez-Gelvez et al. [145]	74	CD68	KP1	<2%, >2%	Inferior EFS
Wang et al. [146]	355	CD163	10D6	>9.5%	Inferior PFS, OS
Cencini et al. [147]	37	CD68 CD163 CD68/CD163	PGM1 10D6 Doublestain (automated system Dako)	Steidl score	No No Inferior PFS (IPI ≥ 2)
Cai et al. [148]	112	CD68	KP1	200/hpf	Inferior PFS

Abbreviations: PFS, progression-free survival; OS, overall survival; hpf, high-power field; R, rituximab; DFS, disease-free survival; NS, not significant; EFS, event-free survival; IPI, international prognostic index.

**Table 3:** The role of tumor-associated macrophages (TAM) as treatment target in hematologic malignancies.

Reference	Treatment	Disease	Mechanism of Action	Type of Study	Results
Benerjee et al. [158]	Trabectedin	CLL	Macrophage killing due to CCL2-CCR2 signalling axis inhibition, antiangiogenic	Leukemic mouse model Human CLL cells	Apoptosis trigger MDSC and TAM depletion. M1 shift Memory T-cells increase Increased mice survival
Cucè et al. [159]	Trabectedin	MM	Macrophage killing due to CCL2-CCR2 signalling axis inhibition, antiangiogenic	Human MM cells	Apoptosis trigger VEGF depletion NK cells upregulation
Polk et al. [74]	CSF-1R signalling inhibitor	CLL	CSF-1R signalling Inhibition	Human CLL cells	NLC depletion Reduced CLL cells Viability
Li et al. [160]	BLZ-945	T-ALL	CSF-1R signalling Inhibition	Mouse model	Inhibition of BMDMs viability, LAMs polarization blocking and depletion. Combination of CSF-1R inhibitor and VCR increased the survival of T-ALL mice.
Komohara et al. [161]	PLX3397	ATLL	CSF-1R signalling Inhibition	ATLL cells	Inhibition of ATLL cell proliferation Apoptosis induction
Edwards et al. [162]	GW-2580, ARRY-382	CLL	CSF-1R signaling Inhibition	Human CLL cells	NLC depletion BCR signaling disruption (together with ibrutinib or idelalisib)
Walker et al. [163]	CXCR4 inhibitor plerixafor	T-ALL	CXCR4/CXCL12 axis inhibition	In vitro Xenograft model	Improved survival (together with ruxolitinib and venetoclax) M1 shift of TAM
McClellan et al. [164]	Exposure to myeloid differentiation promoting cytokines	Ph+ B-ALL	B-ALL blasts reprogram into Macrophage	In vitro Xenograft model	Eliminates B-ALL cells leukemogenicity.
De Vos et al. [165]	Dacetuzumab	R/R DLBCL	anti-CD40 mAb	Phase II study, 46 patients	Disease control (CR/PR/SD) 37%
Liu et al. [166]	Preemptive IFN-α	ALL	TAM reprogramming	47 B-ALL 21 T-ALL (MRD+ after allo-SCT)	M1 shift of TAM, 76% of cases became MRD-
Mancuso et al. [167]	Artesunate	Leukemia	TAM reprogramming JAK2/STAT3 Downregulation	Human monocytes, leukemic cells	Increase in inflammatory monocytes. After contact with monocytes, in vitro apoptosis of leukemic cells increased
Kriston et al. [168]	Lenalidomide	CLL	Abrogation of BMSC survival effect	CLL cells	Abrogation of anti-apoptotic effect of BMSCs on CLL cells.
Chao et al. [169]	Anti-CD47 mAb	ALL	Enabled phagocytosis of tumor cells by TAM	Mouse model	Induces remissions in ALL-engrafted mice
Advani et al. [170]	Anti-CD47 mAb Hu5F9-G4	R/R DLBCL or FL	Enabled phagocytosis of tumor cells by TAM	Phase 1b-2 study	ORR 50% CR rate 36%

Abbreviations: CCL, chemokine ligands with C-C motif; CCR, C-C chemokine receptor; CLL, chronic lymphocytic leukemia; MDSC, myeloid-derived suppressor cells; TAM, tumor-associated macrophages; MM, multiple myeloma; VEGF, vascular endothelial growth factor; NK, natural killer; CSF-1R, colony stimulating factor-1 receptor; NLC, nurse-like cells; ALL, acute lymphoblastic leukemia; BMDM, bone marrow-derived macrophages; LAM, leukemia-associated macrophages; VCR, vincristine; ATLL, adult T-cell lymphoma leukemia; BCR, B-cell receptor; CXCR, chemokine receptor; Ph, Philadelphia; mAb, monoclonal antibody; CR, complete response; PR, partial response; SD, stable disease; IFN, interferon; allo-SCT, allogeneic stem-cell transplantation; MRD, minimal residual disease; JAK, Janus kinase, BMSC, bone marrow stromal cells; DLBCL, diffuse large B-cell lymphoma; FL, follicular lymphoma; ORR, overall response rate.
